# 异基因造血干细胞移植治疗儿童急性淋巴细胞白血病中国专家共识（2022年版）

**DOI:** 10.3760/cma.j.issn.0253-2727.2022.10.001

**Published:** 2022-10

**Authors:** 中华医学会血液学分会干细胞应用学组 中华医学会儿科学分会

急性淋巴细胞白血病（ALL）是儿童时期最常见的恶性肿瘤，目前儿童ALL患者5年总生存率（OS）已超过90％，仍有部分患儿表现为难治或复发，预后不良[1]–[5]。二代测序技术（NGS）、新型靶向药物、新型免疫治疗等诊疗手段不断应用于临床，但异基因造血干细胞移植（allo-HSCT）仍是治疗儿童难治/复发ALL的有效手段[6]。目前我国allo-HSCT相关技术快速发展，allo-HSCT治疗儿童ALL适应证及移植前后处理方案也在不断优化[7]–[8]。为规范我国儿童ALL患者 allo-HSCT适应证、移植前处理、预处理方案、供者选择及移植后原发疾病复发的防治，中华医学会血液学分会干细胞应用学组及中华医学会儿科学分会组织国内相关专家制订本共识。

一、儿童ALL诊断及难治/复发ALL的定义

本共识中儿童年龄定义为0～18岁（本共识临床研究数据主要来源于0～14岁儿童ALL的文献，14～18岁儿童ALL资料较少）。儿童ALL的诊断采用MICM（细胞形态学、免疫学、细胞遗传学及分子遗传学）模式[9]，包含：①骨髓细胞形态学显示原始及幼稚淋巴细胞≥20％；②使用多参数流式细胞术（FCM）进行免疫表型检测，确定白血病类型：主要分为T细胞系及B细胞系两大类；③使用染色体显带技术进行核型分析，明确白血病细胞染色体数目异常及易位、倒位、缺失等结构改变；④分子学检测、NGS检测融合基因及基因突变；⑤脑脊液检查除外中枢神经系统白血病（CNSL）；⑥影像学检查如B超、CT、磁共振（MRI）或PET-CT等除外其他部位的髓外受累。

难治儿童ALL的定义：采用我国协作组标准方案治疗2个疗程未获得完全缓解（CR）；第1次完全缓解（CR_1_）后6个月内复发者；第2次完全缓解（CR_2_）后6个月以上复发但经过标准诱导方案治疗不能获得缓解者；2次或多次复发者；持续存在髓外白血病者。儿童ALL复发的定义：已获得CR的患儿外周血或骨髓再次出现原始细胞>5％或出现髓外疾病[10]–[11]。髓外复发指在中枢神经系统（CNS）、睾丸及其他髓外部位发现形态学证实的白血病细胞。分子学复发：治疗前有遗传学标记及免疫表型特点的患者，治疗后多参数FCM、融合基因等检测由阴性转为阳性且复查仍为阳性者。

二、微小残留病（MRD）的定义及检测

MRD指常规形态学无法发现的白血病细胞[12]。目前儿童ALL主要的MRD检测方法有多参数FCM、实时定量PCR检测特异性融合基因或IgH/TCR基因克隆性重排及NGS检测融合基因或IgH/TCR基因克隆性重排。NGS检测ALL患儿IgH/TCR基因克隆性重排在预测白血病复发方面较FCM更为准确[13]，但目前国内尚未大规模开展此类检测，故建议各移植中心根据自身经验结合PCR技术进一步明确其临床意义。

三、儿童ALL危险度分层

根据复发风险及治疗反应将儿童ALL分为低危组、中危组及高危组，中国各儿童血液肿瘤研究中心/协作组的儿童ALL危险度分层存在微小差异。因危险度分层与移植适应证密切相关，本共识推荐的危险度分层标准见表1。

四、儿童ALL患者allo-HSCT适应证

儿童ALL整体治疗效果较好，但对其中诱导缓解治疗失败、MRD持续阳性或血液学复发的小部分儿童ALL患儿建议行allo-HSCT改善预后，低危组、中危组移植适应证分别见表2、表3，而所有被纳入高危组的患儿均建议allo-HSCT。少数原发诱导缓解失败/复发后再诱导缓解失败的ALL患儿，若后续采用化疗/免疫治疗等手段始终未达CR，建议进入临床研究或姑息治疗、allo-HSCT等。

**表1:** 儿童急性淋巴细胞白血病（ALL）危险度分层

组别	描述
低危组（LR）	符合以下所有条件：
	①年龄1~<10岁
	②WBC<50×10^9^/L
	③无CNSL或睾丸白血病
	④非中/高危组细胞遗传学、分子生物学特征
	⑤诱导治疗结束及巩固治疗前FCM-MRD<1×10^−4^
中危组（IR）	符合以下任何1项或多项：
	①年龄≥10岁
	②初诊最高WBC≥50×10^9^/L
	③有髓外浸润、CNS2、CNS3或睾丸白血病
	④t(1;19)(q23;p13)/TCF3-PBX1
	⑤Ph^+^ ALL
	⑥Ph样ALL
	⑦MEF2D基因重排
	⑧iAMP21
	⑨ZNF384基因重排
	⑩无法归入高危组的T-ALL
	⑪其他不能归入低危组及高危组者
高危组（HR）	符合以下任何1项或多项：
	①诱导治疗结束FCM-MRD≥1×10^−2^，或巩固治疗前≥1×10^−4^
	②亚二倍体（染色体数目≤44或DNA指数<0.8）[14]
	③MLL基因重排阳性且年龄<6个月、WBC≥300×10^9^/L；或伴t（4;11）（q21;q23)/MLL-AF4者
	④TCF3-HLF/t(17;19)(q22;p13)
	⑤中危患儿间期治疗中出现FCM-MRD≥0.01%并经复查确认[10]

注：CNS：中枢神经系统；CNSL：中枢神经系统白血病；T-ALL：急性T淋巴细胞白血病；FCM-MRD：流式细胞术检测微小残留病；Ph：费城染色体。CNS1：同时符合以下3项：①脑脊液中无白血病细胞；②无CNS的临床表现，即无明显与白血病有关的颅神经麻痹；③无CNS的影像学依据。CNS2：符合以下任何1项：①腰穿无损伤（脑脊液不混血，RBC∶WBC≤100∶1）时，脑脊液中WBC≤5个/µl并见到明确的白血病细胞；②腰穿有损伤（脑脊液混血，RBC∶WBC>100∶1）时，脑脊液中见到明确的白血病细胞；③腰穿有损伤且为血性脑脊液，如初诊WBC>50×10^9^/L则归为CNS2。CNS3（即CNSL）：①脑脊液中RBC∶WBC≤100∶1，WBC>5个/µl并以白血病细胞为主或白血病细胞所占比例高于外周血幼稚细胞百分比；②无其他明确病因的颅神经麻痹；③CT或磁共振（MRI）显示脑或脑膜病变并除外其他CNS疾病

**表2:** 低危组儿童急性淋巴细胞白血病（ALL）异基因造血干细胞移植（allo-HSCT）适应证

疾病状态	allo-HSCT适应证
CR_1_期	间期治疗中任意时间点的MRD（FCM或特异性融合基因RQ-PCR法）2次转阳者，根据MRD监测结果推荐allo-HSCT[15]–[16]
CR_2_期	①极早期复发（治疗开始18个月内）及早期复发（诊断18个月以上，但停一线治疗6个月内或治疗开始36个月内）的ALL患儿，建议于CR_2_期行allo-HSCT；晚期（>36个月）骨髓复发患儿达到CR_2_后若MRD持续阳性（FCM-MRD≥10^−4^或融合基因阳性），建议行allo-HSCT ②治疗时间<18个月的髓外复发；治疗时间≥18个月的髓外复发，经过4~8周治疗仍未缓解者；骨髓复发伴髓外复发者[10]
CR_3_期	建议allo-HSCT

注：CR_1_、CR_2_、CR_3_分别为第1、2、3次完全缓解。MRD：微小残留病；FCM：流式细胞术；RQ-PCR：实时荧光定量聚合酶链反应。在查询移植适应证前应重新评估危险度，然后按危险度分层查询对应移植适应证

**表3:** 中危组儿童急性淋巴细胞白血病（ALL）异基因造血干细胞移植（allo-HSCT）适应证

疾病类型	CR_1_期	CR_2_期	CR_3_期
Ph^−^B-ALL	①诱导化疗后FCM-MRD 10^−2^~10^−3^的患儿，各移植中心可根据自身经验监测骨髓MRD水平并作出治疗决策[17]–[18] ②规范化疗≥3个月后FCM-MRD阴性但融合基因阳性的患儿，建议密切监测融合基因定量变化，融合基因定量进行性上升者结合FCM检测结果推荐allo-HSCT[19]–[20]	①极早期复发（治疗开始18个月内）及早期复发（诊断18个月以上，但停一线治疗6个月内，或治疗开始36个月内）的ALL患儿，建议于CR_2_期行allo-HSCT；晚期（>36个月）骨髓复发患儿达到CR_2_后若MRD持续阳性者（FCM-MRD≥10^−4^或融合基因阳性）建议行allo-HSCT ②治疗时间<18个月的髓外复发；治疗时间≥18个月的髓外复发，经过4～8周治疗仍未缓解者；骨髓复发伴髓外复发者[10]	建议allo-HSCT
Ph^+^ B-ALL	①Ph^+^ B-ALL或BCR/ABL融合基因阳性B-ALL患儿标准诱导化疗后未缓解[10] ②规律化疗联合口服TKI（伊马替尼或达沙替尼）治疗3个月，BCR/ABL融合基因定量较初治定量降低不足3个对数级[7] ③规律化疗联合口服TKI无效，BCR/ABL融合基因仍持续上升者，有移植经验的中心可行allo-HSCT或进入临床研究 ④ABL位点存在T315I突变者 ⑤不耐受TKI治疗者	建议allo-HSCT	建议allo-HSCT
T-ALL	强化治疗结束时仍存在髓外白血病病灶者（包括纵隔、淋巴结、中枢神经系统等部位）[21]–[22]	建议allo-HSCT	建议allo-HSCT

注：CR_1_、CR_2_、CR_3_分别为第1、2、3次完全缓解。B-ALL：急性B淋巴细胞白血病；T-ALL：急性T淋巴细胞白血病；Ph：费城染色体；FCM-MRD：流式细胞术检测微小残留病；TKI：酪氨酸激酶抑制剂。在查询移植适应证前应重新评估危险度，然后按危险度分层查询对应移植适应证

五、儿童ALL患者allo-HSCT前处理

（一）降低移植前肿瘤负荷

儿童ALL移植疗效与移植前MRD水平密切相关，故应尽可能降低难治/复发ALL患儿移植前肿瘤负荷[23]。推荐有条件的移植中心在移植前检测MRD后决定桥接allo-HSCT或其他有效治疗方法。

化疗是复发ALL再诱导缓解最常用的疗法，建议使用第一次诱导缓解的方案，在患儿监护人充分知情同意后亦可考虑使用之前未使用过或应用较少的化疗药物，例如BCL-2抑制剂[24]–[25]、硼替佐米[26]、氯法拉滨[27]、奈拉滨[28]–[29]、地西他滨[30]及异环磷酰胺[31]等。

为了诱导儿童难治/复发B-ALL达到CR或消除MRD，可考虑使用嵌合抗原受体T细胞（CAR-T）治疗、CD19-CD3双特异性抗体药物Blinatumomab、人源化CD22抗体免疫毒素Inotuzumab calicheamicin等新型免疫疗法，其中以CD19为靶点的CAR-T细胞治疗及CD19-CD3双抗在诱导难治/复发B-ALL患儿达到CR后桥接allo-HSCT的临床效果较好[32]–[35]。各移植中心可根据患儿条件、免疫疗法可获得情况等开展新型免疫治疗。

难治/复发急性T淋巴细胞白血病（T-ALL）可使用化疗诱导缓解，以CD7、CD5为靶点的CAR-T细胞疗法目前尚处于临床试验阶段。

（二）髓外病灶的处理

移植前应评估髓外受累情况，必要时可考虑使用PET-CT辅助评估髓外病灶。若诱导化疗结束后出现CNSL，可使用三联鞘内注射或全颅脑脊髓放疗达到CR后行allo-HSCT。CAR-T细胞疗法亦可用于治疗儿童B-ALL的CNSL[36]。儿童ALL在移植前一般要求行三联鞘内注射6～8次，若次数不够，可于移植后补齐；移植后至少行1次脑脊液检查（脑脊液常规、生化、甩片、FCM）。移植前有明确CNSL或T-ALL患儿，移植后可视患儿临床情况行脑脊液监测/鞘内注射，鞘内注射累计不超过30次。

若患儿存在睾丸白血病复发，经病理确诊后可考虑行睾丸局部放疗，亦可使用CAR-T细胞治疗。若有其他髓外病灶，可使用化疗、局部放疗、免疫治疗、手术等治疗手段清除病灶后行allo-HSCT。

六、供者选择

儿童难治/复发ALL可选择HLA相合同胞供者；若无HLA相合同胞供者，单倍体供者、非血缘HLA 9/10或10/10相合供者、脐血干细胞均可考虑作为一线选择。单倍体造血干细胞移植（haplo-HSCT）几乎完全解决了供者来源问题，可方便、迅速地开展移植工作，是理想的儿童ALL紧急挽救性移植模式[37]。具体选择供者时要根据患者病情、供者情况、各单位移植经验、移植后供者来源细胞获取情况综合考虑。

非体外去除T细胞儿童haplo-HSCT供者选择推荐顺序为：父亲、母亲（母亲≤45岁的患儿，移植后可考虑加用小剂量环磷酰胺）、非遗传性母亲抗原（NIMA）不合的同胞、非遗传性父亲抗原（NIPA）不合的同胞及其他旁系亲属[38]。体外去除T细胞haplo-HSCT供者选择，要注意选择供者NK细胞对患者细胞具有同种异体反应性的供者[39]。

供者特异性抗人类白细胞抗原抗体（DSA）与haplo-HSCT后植入不良相关。儿童ALL造血干细胞移植患者中抗HLA-Ⅰ类抗原抗体阳性率为10.7％，抗HLA-Ⅱ类抗原抗体阳性率为4.9％，同时具备两类HLA抗原抗体者占2.7％，儿童ALL与儿童骨髓增生异常综合征相比DSA处于较低水平[40]。haplo-HSCT前需筛查DSA水平，若DSA<2 000，无需处理；若DSA 2 000～10 000，各移植中心根据自身经验可采用抗CD20单抗、脐血输注、静注人免疫球蛋白或联合方案等干预方法；若DSA>10 000，建议更换供者[41]。若患者DSA高水平且无合适供者，有经验的移植中心可结合患者具体DSA情况，试用血浆置换、静注人免疫球蛋白、抗CD20单抗等方法降低DSA水平[42]。

脐血也是一个较好的造血干细胞来源，特别是NK细胞异源反应性好的脐血可降低复发率[43]。脐血的选择要根据脐血的有核细胞总数（TNC）、CD34^+^细胞数、供患者HLA位点匹配程度、患者特异性HLA抗体相匹配抗原及病情综合考虑。

七、预处理方案

预处理指患儿在造血干细胞回输前接受的全身放射治疗（TBI）和（或）细胞毒药物及免疫抑制剂的联合治疗。儿童难治/复发ALL的预处理方案包含两个目的：进一步降低肿瘤负荷及免疫抑制以克服HLA屏障。理想预处理方案的选择应基于疾病相关因素（诊断、类型、受累部位及MRD水平等）及患儿相关因素（供者类型、干细胞来源、合并症等）。

在同胞全相合造血干细胞移植（Sib-HSCT）及无关供者造血干细胞移植（UD-HSCT）中，以白消安为基础的预处理方案及以TBI为基础的预处理方案均可采用（表4）。有研究显示，在Sib-HSCT及UD-HSCT中，移植时为CR_1_期患者使用包含分次TBI（FTBI）方案与以白消安为主预处理方案取得的疗效类似，而CR_2_期患者使用含TBI预处理方案则疗效更佳[44]；亦有证据显示，无论CR_1_期还是CR_2_期，在4岁以上儿童ALL患者中包含FTBI预处理方案Sib-HSCT及UD-HSCT组长期生存更优[45]。而以白消安为主的haplo-HSCT预处理方案在儿童ALL中亦取得较好疗效[7],[14]。建议各移植中心在临床实践中根据患儿年龄（至少4周岁以上）、疾病情况、髓外受累程度、肝肾功能、生长发育等条件综合考虑后选择TBI或非TBI预处理方案。TBI分为单次TBI（STBI）及FTBI，STBI总剂量一般不超过12 Gy，目前haplo-HSCT单次建议剂量7.7～8.0 Gy[14]。FTBI具有患者耐受好、可适当提高总剂量、剂量误差小等优点，建议12 Gy或10 Gy分3 d照射[46]。儿童患者白消安剂量应根据年龄、体重计算，但药物代谢个体差异较大。建议有条件的移植中心对儿童ALL移植患者特别是婴幼儿移植患者监测白消安血药浓度以指导临床用药[47]。北京方案（非体外去T细胞）及后置环磷酰胺（PTCY）方案是目前两个主流的haplo-HSCT方案[48]–[49]。北京方案包含TBI及化疗两种预处理方案，实际应用中可根据难治/复发ALL患儿疾病类型、骨髓外受累情况、各脏器功能状态及耐受性进行选择。特别高危的患者可考虑接受强化预处理方案。强化预处理方案一般在经典方案基础上增加一些化疗药物，常用依托泊苷、地西他滨、美法仑、阿扎胞苷、氟达拉滨及塞替哌等（表5）。

**表4:** 儿童急性淋巴细胞白血病（ALL）异基因造血干细胞移植（allo-HSCT）改良预处理方案

预处理方案	适用移植类型	具体方案
经典方案		
Cy/TBI	allo-HSCT	分次TBI 12~14 Gy，−13~ −1 d；环磷酰胺 120 mg·kg^−1^·d^−1^，−6 d、−5 d
Bu/Cy	allo-HSCT	白消安16 mg·kg^−1^·d^−1^（口服）或12.8 mg·kg^−1^·d^−1^（静脉滴注），−7~ −4 d；环磷酰胺120 mg/kg，−3 d、−2 d给药
改良方案		
mBu/Cy	Sib-HSCT	阿糖胞苷2 g/m^2^，−9 d；白消安总量 9.6 mg/kg（静脉滴注），−8 ~ −6 d；环磷酰胺总量3.6 g/m^2^，−5 d、−4 d；MeCCNU 250 mg/m^2^，−3 d口服
mCy/TBI	Sib-HSCT	TBI 770 cGy，−6 d；环磷酰胺3.6 g/m^2^，−5 d、−4 d；MeCCNU 250 mg/m^2^，−3 d口服
mBuCy+ATG	UD-HSCT、CBSCT、haplo-HSCT	阿糖胞苷 2~4 g·kg^−1^·d^−1^，−10 d、−9 d；白消安9.6 mg/kg（静脉滴注），−8~ −6 d； 环磷酰胺3.6 g/m^2^，−5 d、−4 d；MeCCNU 250 mg/m^2^，−3 d口服；兔抗人胸腺细胞免疫球蛋白7.5~10 mg/kg或抗人T细胞兔免疫球蛋白40 mg/kg，−5 ~ −2 d
mCy/TBI+ATG	UD-HSCT、CBSCT、haplo-HSCT	TBI 770 cGy，−6 d；环磷酰胺3.6 g/m^2^，−5 d、−4 d；MeCCNU 250 mg/m^2^，−3 d口服；兔抗人胸腺细胞免疫球蛋白7.5~10 mg/kg或抗人T细胞兔免疫球蛋白40 mg/kg，−5 ~ −2 d
Bu/Cy/Flu	CBSCT[50]	白消安9.6 mg/kg，−9~ −6 d（静脉）；环磷酰胺3.6 g/m，−5 d、−4 d；氟达拉滨120 mg/m^2^，−5 ~ −2 d

注：TBI：全身放射治疗；MeCCNU：司莫司汀；ATG：抗胸腺细胞球蛋白；UD-HSCT：无关供者造血干细胞移植；Sib-HSCT：同胞全相合造血干细胞移植；CBSCT：脐血造血干细胞移植；haplo-HSCT：单倍体造血干细胞移植。CBSCT的ATG剂量可根据各中心实际情况进行调整

**表5:** 儿童急性淋巴细胞白血病（ALL）异基因造血干细胞移植（allo-HSCT）强化预处理方案

预处理方案	适用移植类型	药物名称
TBI/TT/Cy	Sib-HSCT	TBI 12 Gy，−7 ~ −5 d；塞替派10 mg/kg，−4 d；环磷酰胺120 mg/kg，−3 d、−2 d
Cy/VP/TBI	Sib-HSCT	环磷酰胺120 mg/kg，−6 d、−5 d；依托泊苷40 ~ 60 mg/m^2^，−4 d；TBI 12.0~14.5 Gy，−3 ~ −1 d
treosulfan/TT/Flu	Sib-HSCT/UD-HSCT	曲奥舒凡（Treosulfan）：体表面积<0.5 m^2^者10 g·m^−2^·d^−1^，体表面积0.5 m^2^~1 m^2^者12 g·m^−2^·d^−1^，体表面积>1 m^2^者14 g·m^−2^·d^−1^，均为−6 ~ −4 d给药；氟达拉滨150 mg/m^2^，−7 ~ −3 d；塞替派10 mg/kg，−2 d

注：Sib-HSCT：同胞全相合造血干细胞移植；UD-HSCT：无关供者造血干细胞移植；TBI：全身放射治疗

八、儿童ALL移植后复发/MRD阳性的预防及治疗

（一）儿童ALL移植后复发/MRD阳性的预防

1. 预防性改良供者淋巴细胞输注（mDLI）：供者T淋巴细胞免疫耗竭是移植后白血病复发的原因之一，而供者淋巴细胞输注（DLI）有可能逆转免疫耗竭。与传统DLI不同，mDLI指使用粒细胞集落刺激因子（G-CSF）动员的外周血前体细胞、输注后行短程免疫抑制剂预防DLI相关GVHD的mDLI[51]。推荐用于移植前处于未缓解状态的少数患儿（包括原发未缓解、复发未缓解）或CR_3_期ALL患儿。mDLI前要求无复发、无活动性GVHD、无感染及脏器功能衰竭。同胞全相合造血干细胞移植（Sib-HSCT）患者在移植后30 d左右，haplo-HSCT患者在移植后45～60 d进行预防性mDLI。回输动员后的外周血造血干细胞（输注单个核细胞1×10^8^/kg，CD3^+^细胞1×10^6^/kg～1×10^7^/kg），mDLI前原有基础免疫抑制剂不停用，mDLI后原有基础免疫抑制剂维持有效浓度至少6周，若无GVHD则8周减停。6个月后若无MRD及GVHD，可考虑再次mDLI。

2. 预防性靶向药物的应用：推荐伴有费城染色体阳性（Ph^+^）或BCR/ABL融合基因阳性ALL患儿移植后口服TKI以预防复发[7]。移植后1个月评估骨髓缓解状态，若BCR/ABL基因阴性，无需G-CSF应用情况下中性粒细胞绝对计数（ANC）>1.5×10^9^/L、PLT>75×10^9^/L，即可开始全量TKI预防。若BCR/ABL基因阳性，无论血象如何应立即开始口服TKI。Ph^+^ ALL患儿移植后TKI的选择应结合移植前耐药情况及ABL点突变等多因素综合考量，可选用伊马替尼、达沙替尼、普纳替尼等。应用TKI前BCR/ABL基因定量阴性者，建议用药6～12个月；应用TKI前BCR/ABL基因阳性者，建议用药12～24个月。

（二）儿童ALL移植后MRD阳性的治疗

建议移植后定期监测MRD，若发现MRD阳性，应立即干预、治疗。

1. 减/停免疫抑制剂：Sib-HSCT患儿移植3个月后出现MRD阳性：单次MRD指标异常时即停用免疫抑制剂，观察2周，若无GVHD且复查MRD指标仍异常，可开始mDLI（输注前加或不加化疗）；若无GVHD但复查MRD指标恢复正常，可考虑开始干扰素治疗，继续严密监测。

haplo-HSCT患儿移植100 d后出现MRD阳性：首次MRD阳性时免疫抑制剂减半，观察1周后停用，再观察1周，若无GVHD且复查MRD仍阳性，开始mDLI（治疗前加或不加化疗）；若无GVHD但复查MRD指标阴性，可考虑开始干扰素治疗。

2. CAR-T细胞治疗：B-ALL患儿移植后每次骨髓穿刺检查时均应检测FCM-MRD，使用的抗体组合应包含CD19和CD22。若移植后MRD免疫表型显示CD19和（或）CD22阳性，可考虑使用供者来源或自体来源、以CD19或CD22为靶点的CAR-T细胞治疗[52]–[53]。若CAR-T细胞治疗后MRD转阴，后续最佳治疗尚待探讨，建议密切随访观察。

3. CD19-CD3双特异性抗体药物：移植后若MRD免疫表型显示CD19阳性，亦可选择CD19-CD3双特异性抗体药物[54]。

4. 干预性DLI：推荐用于移植后2个月至1年期间MRD转阳的ALL患儿。Sib-HSCT患者移植后2～3个月出现MRD阳性，则维持原有免疫抑制剂，进行DLI（治疗前加或不加化疗）；移植3个月后出现MRD阳性可停用免疫抑制剂，观察2周；若无GVHD且复查MRD阳性，开始DLI（治疗前加或不加化疗）；若无GVHD且MRD转阴，可考虑开始干扰素治疗。

haplo-HSCT患儿移植后2个月至100 d期间出现MRD结果阴性转阳性，可维持原有免疫抑制剂、进行mDLI（治疗前加或不加化疗）。移植100 d后出现MRD结果阴性转阳性，第1次MRD转阳时免疫抑制剂减半至停用，若无GVHD且MRD阳性，可开始mDLI（治疗前加或不加化疗）；若无GVHD且MRD为阴性，可考虑开始干扰素治疗。

mDLI前的化疗剂量不宜过大，主要结合mDLI发挥作用并应考虑移植后患者耐受性。细胞输注时间为化疗停止后2～3个半衰期。环孢素A（CsA）于mDLI前1 d开始应用，Sib-HSCT患者在mDLI后4周内保持有效浓度>150 µg/L，若无GVHD则开始减量，6周减停；haplo-HSCT患者在mDLI后6周内保持有效浓度>150 µg/L，8周停药。若回输后MRD阴性、无GVHD，6个月后可再行mDLI。

（三）儿童ALL移植后血液学复发的治疗

1. 治疗性mDLI：血液学复发的急性T-ALL患者首选化疗+DLI。移植100 d后出现血液学复发，可立刻停用免疫抑制剂并开始化疗+mDLI。B-ALL患儿复发亦可选用mDLI（加或不加化疗）。回输前化疗可采用大剂量甲氨蝶呤（1～2 g/m^2^）、CODP方案（环磷酰胺800 mg·m^−2^·d^−1^，第1、2天；长春新碱1 mg/m^2^，第1天；柔红霉素40 mg·m^−2^·d^−1^，第1、2、3天；泼尼松60 mg/d×7 d）等化疗方案[55]。

2. 治疗性CAR-T细胞治疗：患者血液学复发后应检查复发后白血病细胞免疫表型，若CD19和（或）CD22阳性，可使用CD19-CAR-T细胞治疗或CD22-CAR-T细胞治疗诱导缓解，CR率可达80％以上[56]–[57]，若获得形态学缓解可考虑二次移植或临床试验。

3. CD19-CD3双特异性抗体药物：患者血液学复发后若CD19阳性（特别是移植后3个月内的患儿），可考虑CD19-CD3双特异性抗体药物，可连续使用1～2个周期。

4. 二次移植：患儿复发后使用化疗、CAR-T细胞治疗或双特异性抗体药物达到CR后，根据复发时间早晚、上述治疗后MRD是否转阴并结合患儿身体状况、个人及家属意愿决定是否进行二次移植。应检测HLA区域杂合性缺失（HLA loss），为供者选择提供参考。二次移植方案亦应根据疾病类型、患儿身体状态、脏器功能等综合考虑。

（四）儿童ALL移植后髓外复发的治疗

视病变受累的范围选择局部治疗、全身治疗或联合治疗。局部治疗包括手术切除、鞘内注射及局部放疗，全身治疗包括化疗、DLI、CAR-T细胞治疗及二次移植。建议局部治疗后进行全身治疗如DLI等预防骨髓复发。部分ALL早期CNS复发可在减/停免疫抑制剂的情况下选用鞘内注射化疗药物或使用颅脑脊髓放疗。CAR-T细胞治疗对CNSL及睾丸白血病均有疗效，但若CNS肿瘤负荷高，CAR-T细胞治疗的神经系统毒性不可忽视[58]–[59]。

总之，allo-HSCT是治疗儿童难治/复发ALL的有效手段，复发的预防应贯穿整个移植过程，如此才可进一步改善儿童ALL的长期生存率。
